# Associations between inflammatory and angiogenic proteomic biomarkers, and cardiovascular events and mortality in relation to kidney function

**DOI:** 10.1093/ckj/sfae050

**Published:** 2024-03-01

**Authors:** Barbara Salzinger, Kristina Lundwall, Marie Evans, Josefin Mörtberg, Håkan Wallén, Tomas Jernberg, Thomas Kahan, Pia Lundman, Per Tornvall, David Erlinge, Bertil Lindahl, Tomasz Baron, Melinda Rezeli, Jonas Spaak, Stefan H Jacobson

**Affiliations:** Division of Nephrology, Department of Clinical Sciences, Danderyd Hospital, Karolinska Institute, Stockholm, Sweden; Division of Cardiovascular Medicine, Department of Clinical Sciences, Danderyd Hospital, Karolinska Institute, Stockholm, Sweden; ME Renal Medicine, Department of Clinical Intervention and Technology, Karolinska Institute, Stockholm, Sweden; Division of Nephrology, Department of Internal Medicine, Centre for Clinical Research, County of Vastmanland and Uppsala University, Uppsala, Sweden; Division of Cardiovascular Medicine, Department of Clinical Sciences, Danderyd Hospital, Karolinska Institute, Stockholm, Sweden; Division of Cardiovascular Medicine, Department of Clinical Sciences, Danderyd Hospital, Karolinska Institute, Stockholm, Sweden; Division of Cardiovascular Medicine, Department of Clinical Sciences, Danderyd Hospital, Karolinska Institute, Stockholm, Sweden; Division of Cardiovascular Medicine, Department of Clinical Sciences, Danderyd Hospital, Karolinska Institute, Stockholm, Sweden; Department of Clinical Science and Education, Sodersjukhuset, Karolinska Institute, Stockholm, Sweden; Department of Cardiology, Clinical Sciences, Lund University, Lund, Sweden; Department of Medical Sciences, Cardiology, Uppsala Clinical Research Centre, Uppsala University, Uppsala, Sweden; Department of Medical Sciences, Cardiology, Uppsala Clinical Research Centre, Uppsala University, Uppsala, Sweden; Clinical Protein Science & Imaging, Department of Biomedical Engineering, Lund University, Lund, Sweden; Division of Cardiovascular Medicine, Department of Clinical Sciences, Danderyd Hospital, Karolinska Institute, Stockholm, Sweden; Division of Nephrology, Department of Clinical Sciences, Danderyd Hospital, Karolinska Institute, Stockholm, Sweden

**Keywords:** ACS, biomarkers, kidney function, prognosis

## Abstract

**Background:**

The links between chronic kidney disease (CKD) and the high burden of cardiovascular disease remain unclear. We aimed to explore the association between selected inflammatory and angiogenic biomarkers, kidney function and long-term outcome in patients with an acute coronary syndrome (ACS) and to test the hypothesis that CKD status modifies this association.

**Methods:**

A total of 1293 ACS patients hospitalized between 2008 and 2015 were followed until 31 December 2017. Plasma was collected on days 1–3 after admission. A total of 13 biomarkers were a priori identified and analysed with two proteomic methods, proximity extension assay or multiple reaction monitoring mass spectrometry. Boxplots and multiple linear regression models were used to study associations between biomarkers and kidney function and adjusted standardized Cox regression with an interaction term for CKD was used to assess whether CKD modified the association between biomarkers and major adverse cardiovascular events and death (MACE+).

**Results:**

The concentrations of nine biomarkers—endothelial cell-specific molecule-1 (ESM-1), fibroblast growth factor 23 (FGF-23), fractalkine (CX3CL1), interleukin-1 receptor antagonist (IL-1RA), interleukin-18 (IL-18), monocyte chemotactic protein-1 (MCP-1), placenta growth factor (PlGF), transmembrane immunoglobulin 1 (TIM-1) and vascular endothelial growth factor A (VEGFA)—were inversely associated with kidney function. ESM-1, FGF-23 and TIM-1 showed associations with MACE+. Only FGF23 remained independently associated after adjustment for the other biomarkers (hazard ratio per standard deviation increase 1.34; 95% Bonferroni corrected confidence interval 1.19–1.50). None of the biomarkers showed an interaction with CKD.

**Conclusions:**

The concentrations of 9 of the 13 prespecified inflammatory and angiogenic proteomic biomarkers increased when kidney function declined. Only FGF-23 demonstrated an independent association with MACE+, and this association was not modified by CKD status. These findings further support FGF-23 as an independent prognostic marker in ACS patients with and without CKD.

## Graphical Abstract

**Graphical Abstract:**
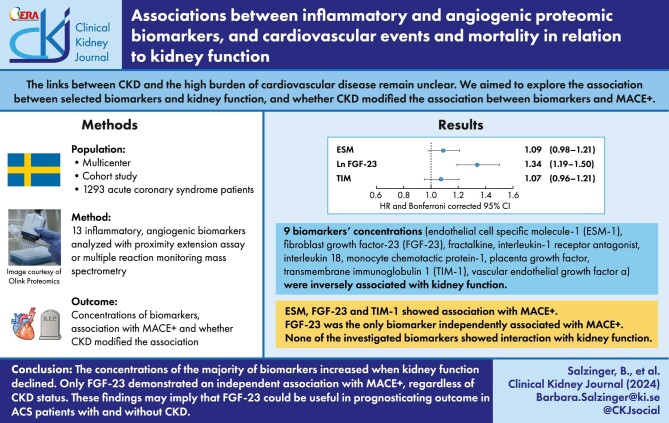

KEY LEARNING POINTS **What was known:**

- The risk for cardiovascular disease (CVD) is greatly increased in patients with chronic kidney disease (CKD) compared with the general population.
- More knowledge is needed about the pathophysiological mechanisms involved in the risk increase.
- Improved knowledge about these mechanisms could help us predict what patients are at greatest risk and focus our efforts and treatments to improve outcomes.

**This study adds:**

- The concentrations of 9 of 13 inflammatory and angiogenic biomarkers thought to be involved in CVD and kidney disease increased when kidney function declined in patients hospitalized for an acute coronary syndrome.
- Three biomarkers (ESM, FGF-23 and TIM-1) showed an association with future cardiovascular events and death, but FGF-23 was the only biomarker independently associated with the outcome.
- CKD status did not modify the association between these biomarkers and future cardiovascular events and death.

**Potential impact:**

- Our results may support FGF-23 as an additional, independent variable to include in future CVD risk stratification models in patients with established CVD, with or without CKD.

## INTRODUCTION

Several large prospective studies have shown that patients admitted with an acute coronary syndrome (ACS), who also have chronic kidney disease (CKD), have inferior short- and long-term prognoses as compared with patients with normal kidney function [1–4]. Many theories have been proposed to explain this unfavourable association between CKD and ACS, including not only traditional cardiovascular risk factors [5, 6], but also the complex biological interplay that evolves with declining kidney function, such as chronic kidney disease mineral and bone disorder (CKD-MBD) [7], vascular calcification, low-grade systemic inflammation [5, 6, 8] and endothelial dysfunction [9], thought to lead to changes in both vascular function and structure [10–12]. However, the importance of each mechanism, how the mechanisms interact and potential additional mechanisms remain to be investigated.

Recent advances in proteomic techniques permit simultaneous analyses of multiple proteins from small samples of blood [13–15], enabling the examination of several potential mechanisms at the same time. Moreover, studies that investigate the association between patterns of biomarkers and outcomes in relation to CKD in ACS patients are scarce, even though this knowledge would facilitate the development of more accurate and clinically important risk prediction models [16].

In a previous study of 1109 patients from the same cohort using a least absolute shrinkage and selection operator (LASSO) regression model, three biomarkers [tumour necrosis factor–related apoptosis-inducing ligand receptor 2 (TRAIL-R2), ovarian cancer–related tumour marker [cancer antigen 125 (CA-125)] and fibroblast growth factor 23 (FGF-23)] among 175 were identified as predictors of all-cause mortality in crude and age-/sex-adjusted analyses, but none of these remained predictive in a fully adjusted model [17]. The LASSO method is a conservative method that can be used to screen large numbers of biomarkers while minimizing the risk for spurious findings at the cost of statistical power. We aimed to further explore the associations between a few carefully preselected putative inflammatory and angiogenic biomarkers and kidney function in patients with ACS and to test the hypothesis that CKD status modified the association between these and the combined outcome major adverse cardiovascular events and death (MACE+).

## MATERIALS AND METHODS

### Study population

This cohort study included hospitalized patients registered in the Swedish Web-System for Enhancement and Development of Evidence-Based Care in Heart Disease Evaluated According to Recommended Therapies (SWEDEHEART) registry and included in the SWEDEHEART biobank. University hospitals in Malmö, Lund, Stockholm and Uppsala participated. Inclusion criteria were hospitalization for suspected ACS between 2008 and 2015 with discharge diagnoses of ST-elevation myocardial infarction (STEMI), non-ST-elevation myocardial infarction (NSTEMI) or unstable angina pectoris. Exclusion criteria were unavailable serum or plasma creatinine and end-stage kidney disease on dialysis.

The patients were provided with oral and written information and signed an informed consent before being included in the study. The Swedish Ethical Review Authority approved the study (2017/759-31) and the study was carried out according to the Declaration of Helsinki.

### Patient characteristics and estimation of kidney function

Patient characteristics including demographics, cardiovascular risk factors, medications and laboratory variables were obtained from the SWEDEHEART registry. This registry has error checking routines, mandatory variables and annual randomly chosen check-ups of entered data with actual patients’ medical records to ensure its high validity [18].

For kidney function assessment, serum or plasma creatinine analysed by either the enzymatic or corrected Jaffe method, both traceable to isotope dilution mass spectroscopy standards, were used. The creatinine at hospital admission was used and assumed to reflect the patients’ baseline creatinine. Previous Swedish data showed that the creatinine level and corresponding estimated glomerular filtration rate (eGFR) at admission for myocardial infarction well reflects patients’ preceding kidney function [19]. Kidney function, as eGFR expressed as ml/min/1.73 m^2^, was estimated from creatinine using the Chronic Kidney Disease Epidemiology Collaboration formula of 2009 [20]. Since data on albuminuria were incomplete, we classified patients into two groups based on eGFR: ≥60 ml/min/1.73 m^2^ was considered as normal kidney function and eGFR <60 ml/min/1.73 m^2^ indicated CKD.

### Analysis of biomarkers

Included patients provided a fasting venous blood sample the mornings of days 1–3 after admission. After centrifugation for 15 min at 2000 *g* at 4°C, the samples were aliquoted and stored at −80°C before analysis. Two different proteomic methods were used: proximity extension assay (PEA) technology [14] or a multiple reaction monitoring (MRM) mass spectrometry method [15].

The PEA technology uses antibodies that bind to the selected proteins, followed by polymerase chain reaction quantification. This method expresses semiquantitative concentrations of the biomarkers transformed to a log_2_ scale. The mean intra-assay coefficient of variation for the panel (Proseek Multiplex CVD I ^96^^X^^96^; Olink Bioscience, Uppsala, Sweden) used in this study was 8% [14].

The MRM method (Lund University, Lund, Sweden) was recently developed and is based on nanoscale liquid chromatographic separation and mass spectrometric detection of unique peptides specific to the target proteins. The method provides the absolute concentration of the measured proteins with a mean. For the assay used in this study, the intra-assay coefficient of variation of 5% [15].

Together, the two methods offered the possibility to analyse 179 proteins. The PEA method used the predefined Olink Proseek Multiplex CVD I ^96^^X^^96^ kit, including 92 proteins presumed to be involved in cardiovascular disease and inflammation. The MRM method was based on an assay analysing 87 proteins thought to be associated with ischaemic heart disease and myocardial infarction [15].

At the start of the project, the available 179 proteins were grouped according to their primary biological function. The categorization was based on information from PubMed (www.pubmed.gov) and Uniprot (http://www.uniprot.org). All biomarkers grouped as inflammation/immunologic activity, angiogenic or kidney damage were further checked for previous knowledge on their role in vascular disease and kidney disease. For 13 biomarkers, there were previous studies supporting their role in vascular and kidney disease, and these were selected a priori for analyses in this study.

### Definition of clinical outcome

Every Swedish citizen has a unique personal identification number. This allowed linking relevant information in the SWEDEHEART registry, the National Patient Registry (including discharge diagnoses for all hospital admissions) and the Swedish Population Registry.

The primary outcome, MACE+, was a composite of the first readmission for myocardial infarction, ischaemic stroke, heart failure or death from any cause. The diagnoses for readmittance according to the International Classification of Diseases, Tenth Revision (ICD) codes were used [myocardial infarction (I21–I22) and ischaemic stroke (I63.0–I63.6) as the main or second diagnosis and heart failure (I50) as the main diagnosis]. Patients were followed until 31 December 2017 and no patient was lost to follow-up.

### Statistical methods

Descriptive statistics are presented as mean [standard deviation (SD)], median [interquartile range (IQR)] or percentages, as appropriate. Biomarkers with a non-normal distribution were log transformed on the natural scale before analyses. The median concentrations of each biomarker were calculated for the group with normal kidney function and for the group with CKD. Non-parametric independent-sample Mann–Whitney U-tests were carried out between groups.

The associations between biomarkers and kidney function (eGFR as a continuous variable) were investigated by multiple linear regression analyses. Adjustments were made for age, sex, body mass index (BMI), smoking habits and diabetes mellitus type 1 and 2. The adjustment variables were chosen a priori.

To explore the association between biomarkers and MACE+, Cox proportional hazard models, adjusted for age, sex, smoking habits, diabetes and CKD, were used. The Schoenfeld residual test was applied to the models and we did not find any violation of the proportional hazard assumptions. The interactions between biomarkers and kidney function were assessed by an interaction term included in the model (biomarker × CKD < or ≥60 ml/min/1.73 m^2^). To study the independent effects of biomarkers, all statistically significant biomarkers from the models with single biomarkers included were used in a final multivariate Cox model. *P*-values <.05 were considered statistically significant, and to consider multiple testing we used a Bonferroni corrected *P*-value (0.05/13 = .0038) when carrying out the Cox models. SPSS software, version 26 (IBM, Armonk, NY, USA) was used for statistical analyses.

## RESULTS

### Study population

**Figure 1:**
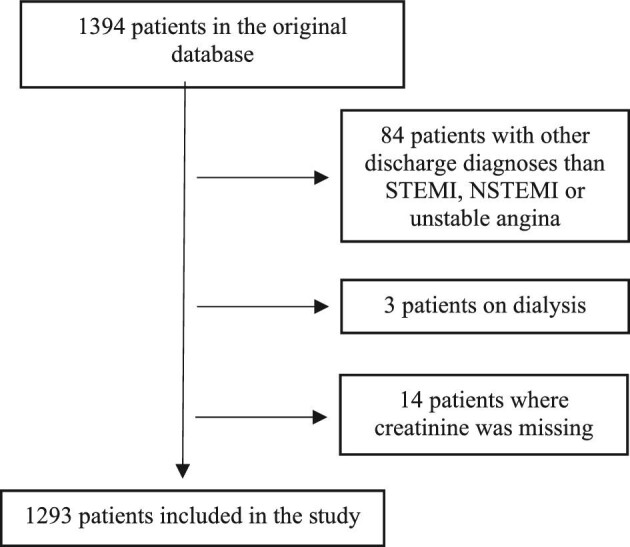
Flow chart of included study population.

The study included 1293 patients (Fig. 1) with a median follow-up of 5.6 years. The median age was 65 years (IQR 58–72), 23% were females and the median eGFR was 85 ml/min/1.73 m^2^ (IQR 67–95; 5th–95th percentile 41–106). Patients with CKD were older, had more cardiovascular risk factors, had a higher proportion of the investigated medications at admission and were also more commonly diagnosed with NSTEMI than STEMI (Table 1).

**Table 1:** Baseline characteristics of study population.

Characteristics	All	eGFR ≥60 ml/min/1.73 m^2^	eGFR <60 ml/min/1.73 m^2^	Missing	*P*-value
Patients, *n*	1293	1079	214		
Demographics					
Age (years), median (IQR)	65 (58–72)	6, (56–70)	75 (69–80)	0	<.01
Female, *n* (%)	295 (23)	233 (22)	62 (29)	0	<.05
Cardiovascular risk factors					
BMI (kg/m^2^), median (IQR)	27 (25–30)	27, (24–29)	27, (25–30)	0	.35
Current/former smokers, *n* (%)	323/440 (28/38)	292/353 (30/37)	31/87 (17/48)	148	<.01
eGFR (ml/min/1.73 m^2^), median (IQR)	85 (67–95)	88 (77–97)	48 (38–55)	0	<.01
Diabetes, *n* (%)	286 (23)	217 (21)	69 (34)	42	<.01
Hypertension, *n* (%)	629 (51)	473 (45)	156 (78)	52	<.01
Previous MI, *n* (%)	217 (19)	155 (16)	62 (33)	125	<.01
Discharge diagnoses index event, *n* (%)					
STEMI	630 (49)	541 (50)	89 (42)	0	<.05
NSTEMI	611 (47)	495 (46)	116 (54)	0	<.05
Unstable angina pectoris	56 (4)	47 (4)	9 (4)	0	.92
Medication on admission, *n* (%)					
ACEi/ARB	364 (31)	263 (27)	101 (53)	134	<.01
Beta-blockers	368 (32)	258 (27)	110 (58)	134	<.01
Calcium channel blockers	205 (18)	143 (15)	62 (33)	134	<.01
Statins	350 (28)	258 (25)	92 (46)	62	<.01
Aspirin	338 (27)	240 (23)	98 (49)	63	<.01

MI: myocardial infarction; ACEi: angiotensin-converting enzyme inhibitor; ARB: angiotensin II receptor blocker.

*P*-value calculated with χ^2^ test or Mann–Whitney U-test as appropriate.

### Associations between biomarkers and kidney function

**Figure 2:**
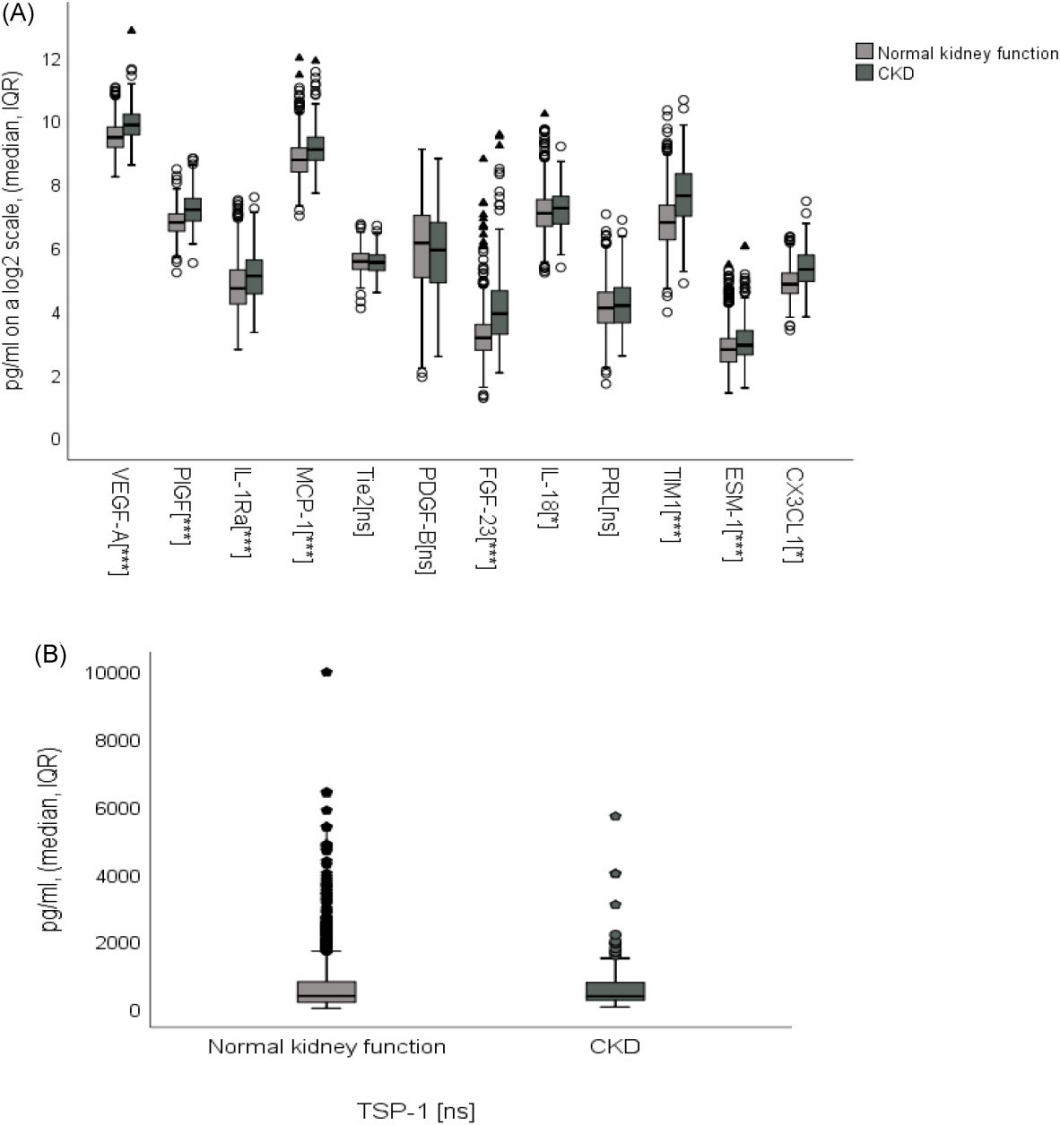
**(A)** Boxplot of PEA biomarker semiquantitative concentrations. Significance was determined by the Mann–Whitney U test. ns: not statistically significant, *P < .05, **P < .01, ***P < .001; o: outlier outside the third quartile + 1.5 × IQR or first quartile − 1.5 × IQR; ▴: extreme outlier outside the third quartile + 3 × IQR or first quartile − 3 × IQR; VEGFA: vascular endothelial growth factor A; PlGF: placenta growth factor; IL-1RA: interleukin-1 receptor antagonist; MCP-1: monocyte chemotactic protein-1; Tie2: angiopoietin-1 receptor; PDGF-B: platelet-derived growth factor subunit B; PRL: prolactin; CX3CL1, fractalkine. **(B)** Boxplot of MRM biomarker quantitative concentrations. Significance was determined by Mann–Whitney U test. ns: not statistically significant; o: outlier outside the third quartile + 1.5 × IQR or first quartile − 1.5 × IQR; ▴: extreme outlier outside the third quartile + 3 × IQR or first quartile − 3 × IQR; TSP-1: thrombospondin 1.

A total of 13 biomarkers, grouped as inflammation/immunologic activity, angiogenic or kidney damage, where previous research has indicated involvement in vascular and kidney disease, were identified (Table 2). Nine of the examined biomarkers were inversely associated with eGFR when comparing biomarker concentrations in patients with and without CKD (Fig. 2a and b, Supplementary Table S1). Adjusted coefficients of determination (*R*^2^) for the multiple linear regression models were low to moderately low (*R*^2^** **= 0.007–0.230) (Table S1).

**Table 2:** Selected biomarkers.

Abbreviation	Full name	Type of analyse	Primary biological function
CX3CL1	Fractalkine	PEA	Inflammatory
ESM-1	Endothelial cell-specific molecule-1	PEA	Angiogenesis
IL-1RA	Interleukin-1 receptor antagonist	PEA	Anti-inflammatory
IL-18	Interleukin-18	PEA	Inflammatory
FGF-23	Fibroblast growth factor-23	PEA	Inflammatory
TSP	Thrombospondin-1	MRM	Coagulation, inflammatory
MCP-1	Monocyte chemotactic protein-1	PEA	Atherosclerosis, inflammatory
PDGF-B	Platelet derived growth factor subunit b	PEA	Angiogenesis
PlGF	Placenta growth factor	PEA	Angiogenesis, atherosclerosis, inflammatory
PRL	Prolactin	PEA	Angiogenesis, coagulation, inflammatory
Tie2	Angiopoietin-1 receptor	PEA	Angiogenesis
TIM-1	Transmembrane immunoglobulin 1/kidney injury molecule 1	PEA	Kidney injury, dysfunction
VEGFA	Vascular endothelial growth factor A	PEA	Angiogenesis

### Association between biomarkers and outcome

**Figure 3:**
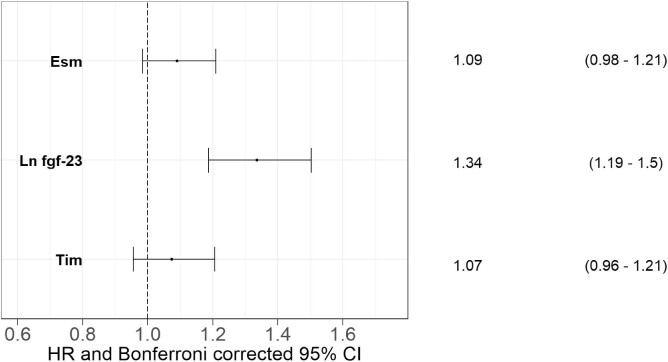
Standardized Cox proportional hazards model association between biomarker and MACE+ (*n* = 1114 total, 377 events). Adjusted for age, sex, diabetes, smoking habits, CKD and for biomarkers with statistically significant HRs in Fig. 3. ln FGF-23: natural logarithm of FGF-23.

Three of the biomarkers, endothelial cell-specific molecule-1 {ESM-1; hazard ratio [HR] per SD increase 1.18 [95% Bonferroni corrected confidence interval (CI) 1.02–1.36]}, FGF-23 [HR per SD increase 1.40 (95% Bonferroni corrected CI 1.19–1.65)] and transmembrane immunoglobulin 1 [TIM-1; HR per SD increase 1.18 (95% Bonferroni corrected CI 1.00–1.40)] showed a significant association with MACE+ in the Cox regression model (Supplementary Fig. S1). None of the included biomarkers showed a significant interaction with kidney function. Only FGF-23 [HR per SD increase 1.34 (95% Bonferroni corrected CI 1.19–1.50)] was shown to have an independent association to MACE+ in the final multivariate Cox model including all three significant biomarkers (Fig. 3).

## DISCUSSION

In this cohort study of 1293 patients with ACS, we showed that 3 of 13 selected putative inflammatory and angiogenic biomarkers (i.e. ESM-1, FGF-23 and TIM-1) were associated with MACE+ in patients hospitalized with ACS. However, FGF-23 was the only biomarker remaining independently associated with MACE+ in a multivariate model. Second, we could not, in our material, show that the association between FGF-23 and MACE+ was significantly modified by kidney function.

In our study (Supplementary Fig. S2), as well as in other previous studies of patients admitted with myocardial infarction, CKD consistently determined a poorer prognosis [21, 22]. The mechanisms underlying this association have not been fully elucidated, but clinical and laboratory features included in CKD-MBD have repeatedly been proposed to play a significant role [6, 7, 23–25]. FGF-23 is a bone-derived hormone that is counterregulatory for phosphate and active vitamin D and thereby maintains phosphorus balance even in conditions with phosphorus excess, such as in CKD [26, 27]. FGF-23 also augments in other conditions often associated with CKD, such as diabetes [24], anaemia, iron deficiency [27] and inflammation [26], indicating that its regulation probably is multifactorial. Clinical studies show that increasing FGF-23, consistently and independent of traditional risk factors, is associated with increased risks of arterial stiffness, left ventricular hypertrophy, heart failure, myocardial infarction, stroke and cardiovascular mortality across a wide range of levels of kidney function [23, 25, 28].

As mentioned in the introduction, a previous study from the same cohort using LASSO identified TRAIL-R2, CA-125 and FGF-23 among 175 biomarkers as predictors of all-cause mortality in crude and age-/sex-adjusted analyses [17], but none of the three biomarkers remained predictive in the fully adjusted models [17]. Another exploratory study from the same cohort, but with a shorter follow-up (3.2 versus 5.6 years) used random forest analyses to identify which biomarkers best predicted CKD. Although not part of the primary analysis, FGF-23 was found to be an important biomarker for all-cause mortality and hospitalization for heart failure in crude models [29]. In this study we expand the present knowledge by showing that FGF-23 has a strong association with long-term MACE+ and that this association remains when adjusting not only for age and sex, but also for diabetes, smoking and CKD. Additionally, none of the previous studies investigated the potential interaction of biomarkers and kidney function in relation to outcome. We analysed this relationship and did not find that the association between FGF-23 and MACE+ was modified by kidney function.

Other studies [23, 30] have demonstrated a significant interaction between FGF-23 and kidney function regarding the risk of cardiovascular death, all-cause mortality or incident heart failure. One possible reason behind the discrepancy in relation to our findings may be that our study population at inclusion already had a high cardiovascular burden. All included patients had manifest cardiovascular disease, whereas in the two other studies only 27–29% of the patients had a prior cardiovascular event [23, 30]. This might suggest that with more advanced cardiovascular disease the interaction between FGF-23 and CKD regarding cardiovascular outcome attenuates. Other reasons that also might have influenced the different findings include slightly better kidney function in our study cohort (mean eGFR 80 ml/min/1.73 m^2^ versus 71–74 ml/min/1.73 m^2^ in the two other cohorts) [23, 30] and longer follow-up time in the two other studies (median 9.7–10.5 years [23, 30] versus 5.6 years).

It is tempting to speculate that it could be of clinical value to measure FGF-23 in ACS patients to try to better assess future cardiovascular risk. Since there currently is no consensus whether the relationship between FGF-23 and cardiovascular events is causal or not [24, 25], the effects of FGF-23-lowering therapies, such as burosumab, in ACS patients remain speculative [26, 31].

In the present study, ESM-1 and TIM-1 were related to MACE+ in adjusted analyses, but not independent of other biomarkers, and not related to kidney function. ESM-1 is a proteoglycan mainly secreted by activated endothelial cells [32, 33], which is thought to be implicated in angiogenesis, vascular remodelling, inflammation and atherosclerosis. TIM-1 is a transmembrane glycoprotein that participates in the regulation of systemic immune reactions [34]. TIM-1 is highly upregulated in kidney tubular epithelial cells and is considered a sensitive and specific marker for early acute kidney injury [35]. Elevated levels in urine have been implicated in CKD progression, in chronic heart failure and in cardiorenal syndrome [36, 37].

This study has strengths and weaknesses. The patients’ characteristics and diagnoses were retrieved from the SWEDEHEART registry. An evaluation study showed that the accuracy between the registry and medical records was 96% [18]. The study cohort was relatively large, with no patients lost to follow-up. However, when generalizing and interpreting results it needs to be taken into consideration that we only included patients with already manifest cardiovascular disease. In addition, our selected cohort was younger, had fewer comorbidities and showed a higher proportion of STEMI in relation to NSTEMI and unstable angina pectoris compared with the Swedish ACS population in general. The cohort also included few patients with severe kidney disease, which might impact the findings.

The PEA and MRM methods permitted high-quality, simultaneous analyses of a large number of cardiovascular biomarkers normally difficult to analyse. We only included a subset of 13 biomarkers thought to be associated with inflammation and angiogenesis and where we could find previous research with a connection to kidney function or vascular disease. The primary biological function of the biomarkers was assessed by using Uniprot and PubMed, which are mainly based on *in vitro* data. We did not confirm the pathological pathways between elevated biomarkers and occurrence of cardiovascular events. Future research and knowledge might show that there are additional biomarkers involved that we left out. Potentially the levels of biomarkers might also be influenced by the different timing of collection (days 1–3 after admittance). In addition, the use of a single measure of biomarkers is problematic in determining their association with MACE+. More biomarker concentrations over time would have strengthened the analyses.

The analysis of the data was adjusted for several confounders, but there might be unknown additional potential confounders not adjusted for, i.e. residual confounding. Furthermore, we have not adjusted for albuminuria and medications due to a lack of complete data. We outlined missing data in Table 1, but did not carry out any separate missing data analyses.

Our study does not include any predictive analyses but focuses on potential associations. These potential associations will need to be further investigated in future studies.

## CONCLUSION

In conclusion, the concentrations of 9 of 13 investigated biomarkers were inversely associated with kidney function. Three biomarkers (ESM, FGF-23 and TIM-1) showed an association with MACE+. FGF-23 was the only biomarker independently associated with MACE+. None of the investigated biomarkers showed an interaction with CKD status.

A validated atherosclerotic cardiovascular disease risk stratification tool for patients with CKD using clinically available variables and novel biomarkers has recently been presented [16]. Our findings suggest that it could be of value to add FGF-23 to future similar equations for patients with established cardiovascular disease, with or without CKD. Before establishing FGF-23 as a target for improving outcomes in patients with ACS, more knowledge about the pathophysiology of FGF-23-induced disease is required.

## Supplementary Material

sfae050_Supplemental_File

## Data Availability

The data underlying this article will be shared upon reasonable request to the corresponding author, if approved by the Swedish Ethical Review Authority.
