# Supplementary material for: Associations between inflammatory and angiogenic proteomic biomarkers, and cardiovascular events and mortality in relation to kidney function
Source: Clin Kidney J. 2024 Mar 1;17(3):sfae050. doi: 10.1093/ckj/sfae050 (PMC10959071; doi:10.1093/ckj/sfae050)
Supplement: sfae050_Supplemental_File [file sfae050_supplemental_file.docx]

SUPPLEMENT

**Table S1:** Multiple linear regression analyses

| **Biomarker** | **Adjusted coefficient of determination** | **β­_eGFR_** | **CI_eGFR_** | **P-value_eGFR_** |
| --- | --- | --- | --- | --- |
| CX3CL1 | 0.138 | 0.007 | -0.008 - -0.005 | <0.01 |
| ESM-1 | 0.054 | -0.001 | -0.003 – 0.001 | 0.354 |
| IL-1Ra | 0.115 | -0.002 | -0.005 – 0.001 | 0.171 |
| IL-18 | 0.029 | -0.001 | -0.003 - 0.002 | 0.487 |
| Ln FGF -23 | 0.218 | -0.004 | -0.005 - -0.004 | <0.01 |
| Ln TSP | 0.007 | 0.003 | 0.000 – 0.007 | 0.086 |
| MCP -1 | 0.098 | -0.005 | -0.008 - -0.003 | <0.01 |
| PDGF -B | 0.011 | 0.006 | 0.002 – 0.011 | <0.05 |
| PlGF | 0.193 | -0.006 | -0.008 - -0.005 | <0.01 |
| PrRL | 0.045 | -0.001 | -0.003 - 0.002 | 0.670 |
| Tie2 | 0.044 | 0.002 | 0.000 - 0.003 | <0.05 |
| TIM1 | 0.230 | -0.010 | -0.013 - -0.007 | <0.01 |
| VEGF -A | 0.138 | -0.007 | -0.009 - -0.005 | <0.01 |

eGFR as a continuous variable, adjustments were made for age, sex, BMI, smoking habits and diabetes. β, beta coefficient, CI, confidence interval, BMI, body mass index; CX3CL1, fractalkine; ESM-1, endothelial cell specific molecule-1; IL-1Ra, interleukin-1 receptor antagonist; IL-18, interleukin 18; ln FGF-23, natural logarithm fibroblast growth factor-23¸ ln TSP-1, natural logarithm thrombospondin 1; MCP, monocyte chemotactic protein-1; PDGF-B, platelet derived growth factor subunit b; PlGF, placenta growth factor; PRL, prolactin; Tie2, angiopoietin-1 receptor; TIM1. transmembrane immunoglobulin 1; VEGF-A, vascular endothelial growth factor a

**Figure S1:** Standardized cox proportional hazard models, association between biomarker and MACE+


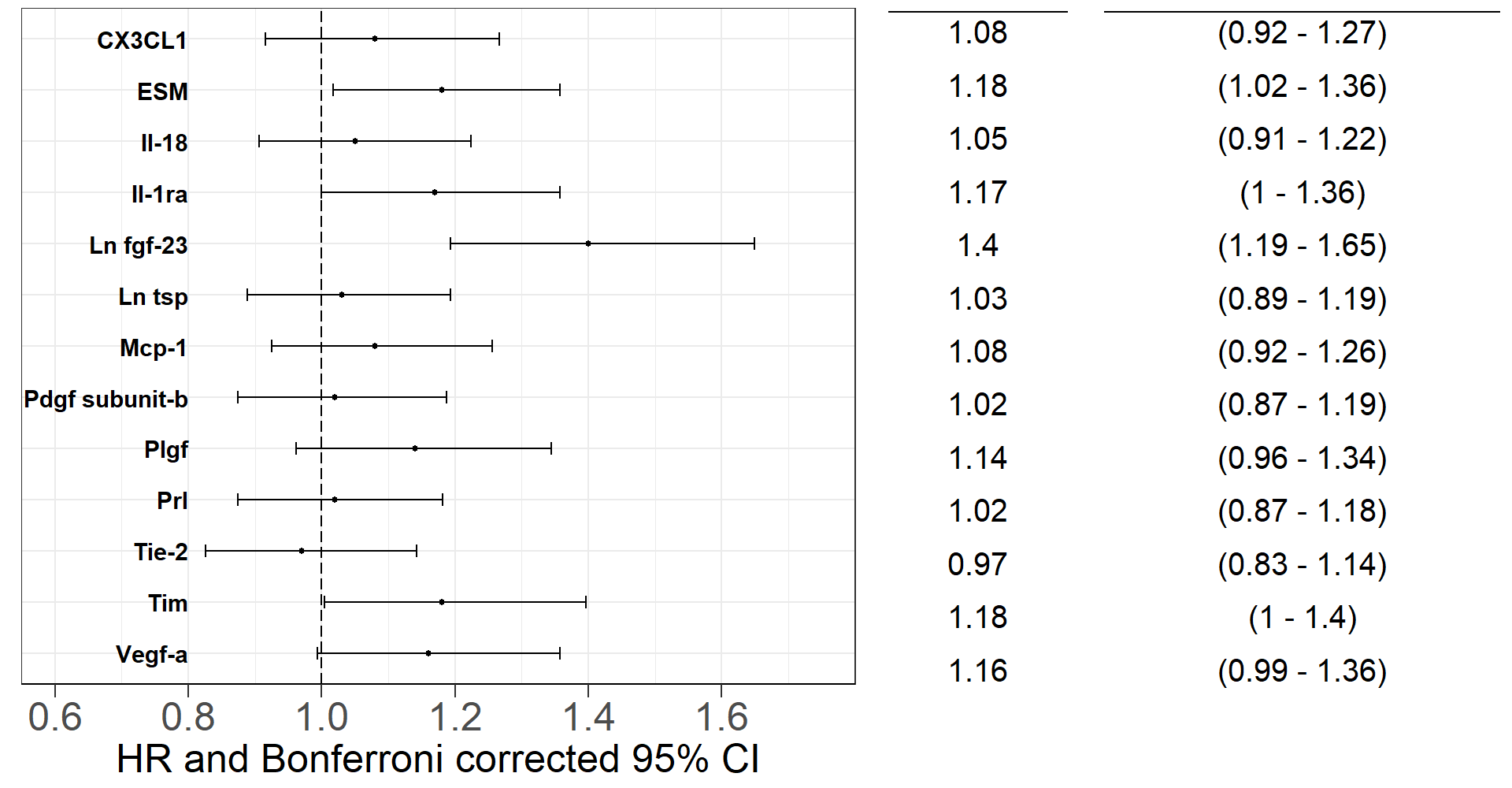


Ln TSP-1: n 1136 total, 386 events, other biomarkers n 1114 total, n 377 events. Adjusted for age, sex, diabetes, smoking habits and CKD. CKD, chronic kidney disease; MACE+, major cardiovascular events and death; CX3CL1, fractalkine; ESM-1, endothelial cell specific molecule-1; IL-1Ra, interleukin-1 receptor antagonist; IL-18, interleukin 18; ln FGF-23, natural logarithm fibroblast growth factor-23¸ ln TSP-1, natural logarithm thrombospondin 1; MCP, monocyte chemotactic protein-1; PDGF-B, platelet derived growth factor subunit b; PlGF, placenta growth factor; PRL, prolactin; Tie2, angiopoietin-1 receptor; TIM1. transmembrane immunoglobulin 1; VEGF-A, vascular endothelial growth factor a; HR, hazard ratio; CI, confidence intervals

**Figure S2:** Kaplan-Meier survival curves for MACE+


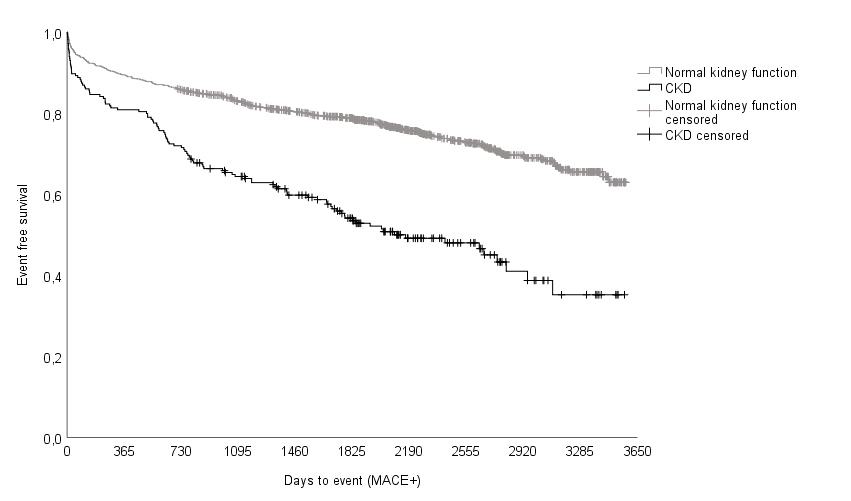


Log rank p-value<0.01. MACE+ major cardiovascular events and death; CKD chronic kidney disease

**Figure S3:** Kaplan-Meier survival curves for MACE+


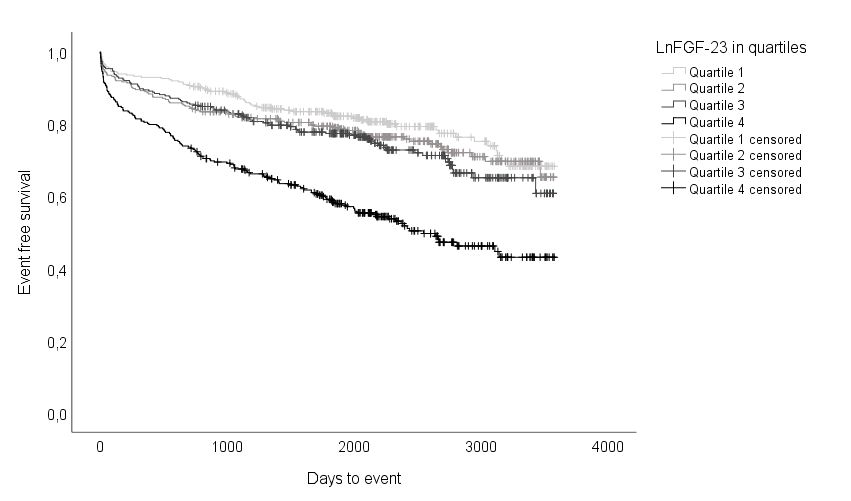


Log rank p-value<0.01. MACE+ major cardiovascular events and death; FGF-23 fibroblast growth factor 23
